# Optically-Induced Neuronal Activity Is Sufficient to Promote Functional Motor Axon Regeneration *In Vivo*

**DOI:** 10.1371/journal.pone.0154243

**Published:** 2016-05-06

**Authors:** Patricia J. Ward, Laura N. Jones, Amanda Mulligan, William Goolsby, Jennifer C. Wilhelm, Arthur W. English

**Affiliations:** 1 Department of Cell Biology, Emory University School of Medicine, Atlanta, Georgia, United States of America; 2 Department of Psychology, College of Charleston, Charleston, South Carolina, United States of America; University of Pennsylvania, UNITED STATES

## Abstract

Peripheral nerve injuries are common, and functional recovery is very poor. Beyond surgical repair of the nerve, there are currently no treatment options for these patients. In experimental models of nerve injury, interventions (such as exercise and electrical stimulation) that increase neuronal activity of the injured neurons effectively enhance axon regeneration. Here, we utilized optogenetics to determine whether increased activity alone is sufficient to promote motor axon regeneration. In *thy-1-ChR2/YFP* transgenic mice in which a subset of motoneurons express the light-sensitive cation channel, channelrhodopsin (ChR2), we activated axons in the sciatic nerve using blue light immediately prior to transection and surgical repair of the sciatic nerve. At four weeks post-injury, direct muscle EMG responses evoked with both optical and electrical stimuli as well as the ratio of these optical/electrical evoked EMG responses were significantly greater in mice that received optical treatment. Thus, significantly more ChR2+ axons successfully re-innervated the gastrocnemius muscle in mice that received optical treatment. Sections of the gastrocnemius muscles were reacted with antibodies to Synaptic Vesicle Protein 2 (SV2) to quantify the number of re-occupied motor endplates. The number of SV2+ endplates was greater in mice that received optical treatment. The number of retrogradely-labeled motoneurons following intramuscular injection of cholera toxin subunit B (conjugated to Alexa Fluor 555) was greater in mice that received optical treatment. Thus, the acute (1 hour), one-time optical treatment resulted in robust, long-lasting effects compared to untreated animals as well as untreated axons (ChR2-). We conclude that neuronal activation is sufficient to promote motor axon regeneration, and this regenerative effect is specific to the activated neurons.

## Introduction

More than 200,000 new, trauma-related nerve injuries occur annually in the United States [1, 2], and an estimated 20 million people are currently affected by some form of peripheral neuropathy according to the National Institutes for Neurological Disorders and Stroke (ninds.nih.gov). Identifying appropriate therapies for these patients could substantially improve functional recovery for patients with traumatic or iatrogenic injuries and delay the functional declines that occur with other forms of peripheral neuropathy. For traumatic nerve injuries (the most common type), surgical repair (primary repair or autograft) is the current gold standard for patients [3].

Axons in peripheral nerves have the ability to regenerate, but the process is complex and very slow. In humans, the achievement of maximal functional recovery can require years, and recovery is very poor [4, 5]. Our lab has previously demonstrated that as little as two weeks of exercise enhances axon regeneration and, over a period of months, enhances muscle re-innervation and reflexes in rats [6]. Rehabilitative approaches for patients have been advocated as previously reviewed [7, 8]. However, patients are often immobilized to prevent nerve tension following a repair. To circumvent this issue, an alternative approach to exercise-based therapies that is being tested clinically is to increase neuronal activity via electrical stimulation applied directly to the injured nerve at the time of repair. Experiments utilizing electrical stimulation in rats resulted in enhanced axon regeneration, and antidromic activation (stimulus evoked action potential propagation toward the central nervous system) was found to be required for this enhancement [9]. Although the pioneering use of electrical stimulation to treat patients recovering from carpal tunnel compression nerve injuries has been effective in humans [10, 11], more research is required to determine how best to implement and tailor this treatment for a very heterogeneous population of patients (e.g., age, sex, fitness level, and health status) with nerve injuries (e.g., proximal versus distal or presence of a gap).

Of particular interest is whether increased neuronal activation is sufficient, by itself, to enhance axon regeneration in cut nerves. The well-established electrical stimulation treatment paradigm (stimulation at 2.5x muscle twitch threshold) activates many axons within the treated nerve. In Al-Majed et al. (2000), the authors cleverly applied tetrodotoxin to the whole femoral nerve to block the antidromic electrical activation of axons, and the results indicate that the stimulation of neuronal cell bodies (not the orthodromic stimulation of the axon/muscle) was necessary for enhanced axon regeneration [9]. However, it is unknown if stimulating a portion of the axons within a motor pool can affect unstimulated motoneurons within that motor pool. By utilizing optogenetics, we were able to address this question. In *thy-1-ChR2/YFP* mice, a subset of motoneurons with axons in peripheral nerves express the light-sensitive cation channel, channelrhodopsin (ChR2). These neurons can be activated selectively, via application of the appropriate wavelength of light to their axons, without affecting other ChR2- axons within the selected nerve. This transgenic mouse provided a unique model that was well-suited to investigate the sufficiency and specificity of neuronal activation following nerve transection. Using this model, we demonstrate that increased neuronal activity is sufficient to enhance axon regeneration after peripheral nerve injury. Moreover, we demonstrate that an acute application of neuronal activity enhanced muscle re-innervation, restored evoked muscle EMG activity (M responses), and promoted the reformation of neuromuscular junctions one month later. The increased activity within the selected ChR2+ neurons did not significantly promote the enhanced regeneration of neighboring neurons (within the same motor nucleus) that were not activated (ChR2-). We interpret these results to indicate that as little as one hour of increased neuronal activity has long-lasting effects on both axon regeneration and muscle re-innervation, and this effect is specific to the activated motoneurons. Clinically, brief electrical stimulation at the time of nerve repair that results in the direct activation of the axotomized neurons should be considered to enhance long-term functional recovery [12].

## Materials and Methods

### Animals

All procedures were approved by the Institutional Animal Care and Use Committee of Emory University (protocol #2003261) and conformed to the Guidelines for the Use of Animals in Research of the Society for Neuroscience. All mice used were on a C57BL/6J background, were backcrossed for at least six generations, and expressed the ChR2/YFP construct under the direction of the *thy-1* promoter (http://jaxmice.jax.org/strain/007612.html) [13]. The mice were group housed (12:12 light:dark cycle) with ad libitum access to food and water. Experiments were conducted on male mice (at least 2 months old) weighing between 18–28 grams. A mixture of ketamine (80 mg/kg) and xylazine (10 mg/kg) was used to induce anesthesia. Additional ketamine was administered as required throughout the surgical procedure. No additional analgesics were administered post-operatively. In *thy-1-ChR2/YFP* mice, the sciatic nerve was exposed and placed on a small rectangle of SILASTIC film (Dow Corning 501–1) and secured using 8 µl of fibrin glue: a mixture of fibrinogen, fibronectin, and thrombin (Sigma-Aldrich, St. Louis, MO) [14, 15]. All surgeries were performed bilaterally.

### Treatments

**Fig 1:**
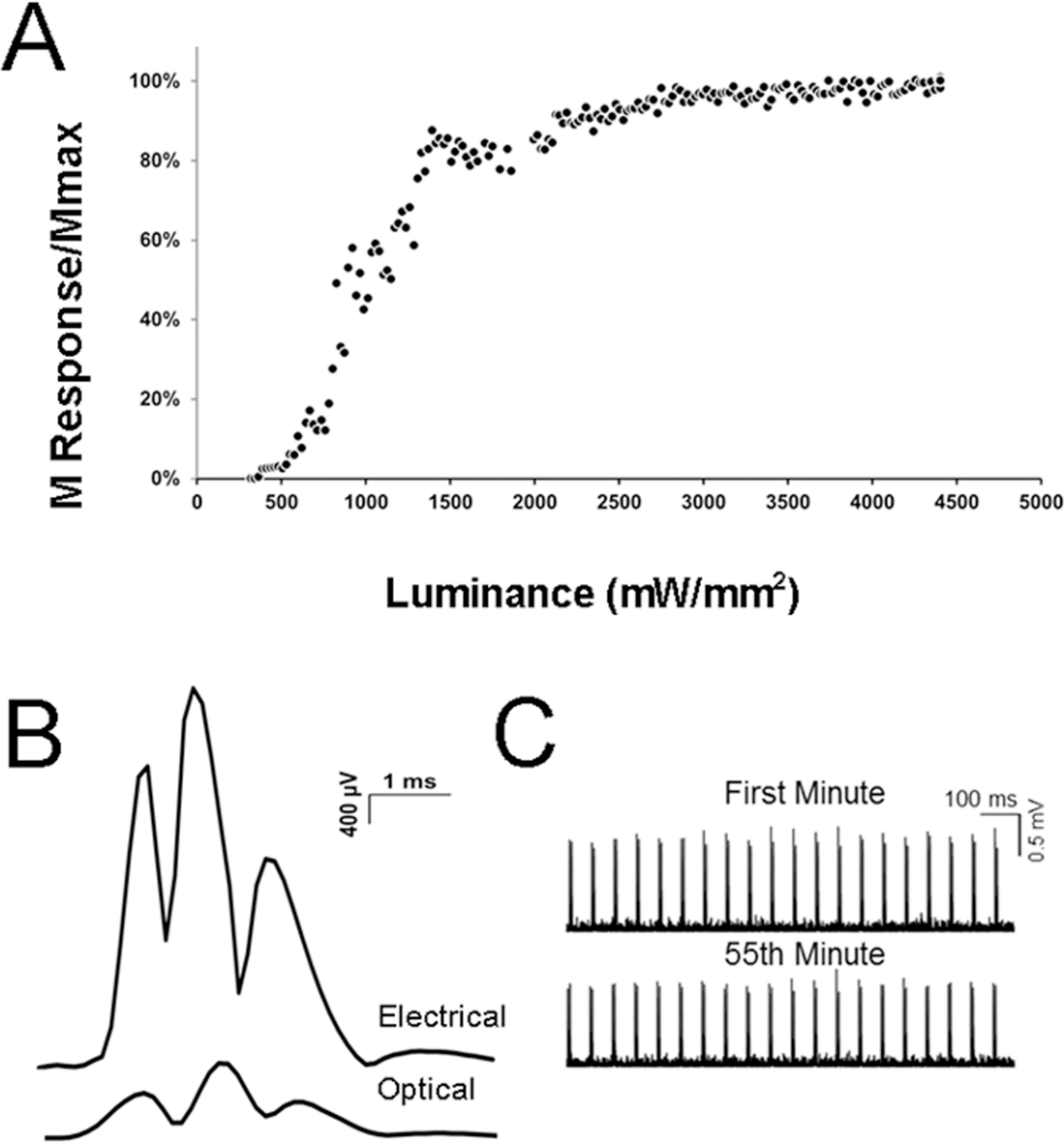
Electromyography. A. The magnitude of the direct muscle (M) EMG responses evoked optically. The M response amplitudes always reached 100% prior to maximizing the laser output. Optical treatments were performed at a supramaximal intensity (similar to electrical stimulation paradigms) B. The magnitude of the direct EMG (M) response evoked electrically (top) or optically (bottom). A subset of axons within the sciatic nerve are ChR2+. Thus, the maximum electrically-evoked M responses were always larger than the maximum optically-evoked M responses. C. EMG responses to a continuous train of optical activations. Muscles will follow up to 20 Hz trains of blue light pulses during an hour of optical treatment.

The mice were randomly assigned to one of three groups (Table 1): intact; transected and untreated; and transected and optical treatment for 1 hour (1 ms, 20 Hz). The groups were assessed at three and four weeks post-transection, as described for the different experiments above (Table 1). For optical treatments, 1 ms duration pulses of blue light (473 nm) were shown directly onto the nerve via a fiber optic cable (200 µm core diameter, 0.39 NA, Thor Labs, Inc., Item number M38L01). The fiber optic cable was positioned with a table-top clamp and maintained gentle contact directly with the sciatic nerve. The optical fiber was attached to a custom built device consisting of a laser LED and a collimator that delivered light pulses at 473 nM wavelength under computer control of intensity and duration. The maximum luminance output of this device was approximately 4500 mW-mm^-2^. This laser device was built in our laboratory. Details are available at web.stanford.edu/group/dlab/optogenetics/hardware.html. Prior to nerve injury in *thy-1-ChR2/YFP mice*, each light pulse caused a visible muscle twitch that was monitored via fine wire electromyography (described below). The amplitude of these optically evoked M responses increased with increasing luminance and reached a maximum typically at about 2600 mW-mm^-2^ (Fig 1A). These M responses are evidence that motor axons were directly activated by light in the *thy-1-ChR2/YFP* mice; in wild type litter mates, no twitches or M responses can be evoked optically. In these experiments, the M response was utilized as an outcome measure, but muscle activation is not required for the enhancing effect of stimulation [9]. We have also recorded light-evoked potentials from the sciatic nerve of mice in which ChR2 is expressed only in motoneurons. These antidromic potentials (recorded proximal to the optical fiber) also support the previous finding that neuronal cell body activation is required for the enhancing effect of optical stimulation on axon regeneration (unpublished data). Optical treatment consisted of 1 hour of supramaximal continuous stimulation (1 ms light pulses, 20 Hz). As previously reported [16] for the *thy-1-ChR2/YFP* mice, muscle responses were evoked consistently at the 20 Hz stimulation rate (Fig 1C).

**Table 1:** Summary of Experiments.

Experiment Name	Time	Treatment	# of cases
Electromyography	3 weeks	Optically treated	17
		Untreated	10
	4 weeks	Optically treated	10
		Untreated	9
Retrograde Labeling (regenerated at least 4 mm)	3 weeks	Optically treated	5
		Untreated	4
Retrograde Labeling (re-innervated gastrocnemius)	4 weeks	Optically treated	6
		Untreated	6
Neuromuscular Junction Re-innervation	3 weeks	Optically treated	6
		Untreated	4
	4 weeks	Optically treated	3
		Untreated	3

### Electromyography Experiments

In these experiments, the entire sciatic nerve was cut and repaired by direct anastomosis and secured with fibrin glue, as described above. The extent of axon regeneration and muscle re-innervation was investigated at different post-injury times electrophysiologically. In ketamine/xylazine anesthetized *thy-1-ChR2/YFP* mice, the right sciatic nerve was exposed via blunt dissection and an electrical stimulating cuff was placed around the whole sciatic nerve. This bipolar stimulating electrode was assembled from a short length of Silastic tubing and stranded stainless steel microwire, AWG size 40 (Cooner Wire, Chatsworth, CA; part number AS631). In post-injury animals, the cuff was placed proximal to the injury site. Fine wire EMG electrodes (California Fine Wire Company, Grover Beach, CA; Stablohm 800 A, material number cfw-100189) were placed into the gastrocnemius muscle using a 25G hypodermic needle. Care was taken to place the electrodes in a common location in the muscle in each animal at each time studied. Electrically evoked EMG activity was recorded from these electrodes in response to sciatic nerve stimulation (0.1 ms pulses). These short latency direct muscle (M) responses are produced by motor axon activation of muscle fibers. Stimulus intensity was incrementally increased until a maximal amplitude M response was recorded (i.e., the response plateaued, see Fig 1A). Stimuli were delivered no more often than once every three seconds to minimize any muscle fatigue. This process was then repeated using a fiber optic cable attached to a 473 nM wavelength laser to obtain the maximal optically evoked M response. The average rectified voltages in a defined M response time window were recorded for each treatment group (untreated and optically stimulated). The M responses were recorded prior to and at three and four weeks following nerve transection and repair.

### Retrograde Labeling Experiments to Assess Axon Regeneration

Immediately following recordings in the three week post-transection and repair mice, to determine the number of motoneurons whose axons successfully regenerated at least four mm into the distal nerve segment, each repaired nerve was transected again four mm below the original transection site. Crystals of dextran amines (10,000 MW, ﬁxable) conjugated Alexa Fluor 555 (Invitrogen, Carlsbad, CA) were applied to the nerve stump [17]. Following a one hour nerve soak, the entire surgical site was washed three times with saline; the surgical wounds were closed; and the animals were returned to their cages for 3–4 days to allow for retrograde transport of the tracer.

### Retrograde Labeling Experiments to Assess Muscle Re-innervation

Immediately following the recordings at four weeks post-transection and repair, a separate retrograde-labeling experiment was performed utilizing tracer injections into muscle targets of the regenerating axons. This approach was assumed to result in retrogradely-labeled motoneurons whose axons had regenerated and re-innervated muscle fibers, whereas the nerve soaking method described above labels all motoneurons whose axon had regenerated four mm and is therefore assumed to be a measure of axon regeneration. Soaking the entire sciatic nerve results in a greater number of retrogradely-labeled motoneurons compared to a tracer injection into a muscle regardless of the time point studied. A total of two µl of Cholera Toxin Subunit B conjugated to Alexafluor 555 (Ctb555) (Life Technologies, Grand Island, NY, catalog number C-34776) was injected bilaterally into the lateral and medial gastrocnemius muscles using a Hamilton syringe. The skin and fascia overlying the medial and lateral gastrocnemius muscles were incised, and 0.5 µl Ctb555 was injected into two sites in each muscle, to minimize tracer leakage. Thus, the lateral and medial gastrocnemius muscles received one µl each. The needle was held in place for 30 seconds prior to withdrawal, and sterile gauze was applied to the needle site for 30 seconds to minimize potential tracer leakage. The animals were returned to their cages for 3–4 days to allow for retrograde transport of the dye.

All mice were euthanized 3–4 days after either nerve soak or muscle injection. All animals were deeply anesthetized with pentobarbital (150 mg/kg, i.p.) and perfused transcardially with 0.9% saline and 4% periodate-lysate-paraformaldehyde fixative [18]. The L3-5 spinal cord segments were explanted and stored in 20% sucrose at 4°C for cryoprotection. The spinal cords were then serially sectioned in the transverse plane at 20 µm and all sections were placed onto slides. High resolution RGB images (20X) were obtained with a Leica DM6000 upright microscope, a low-light camera, and Simple PCI software (Hamamatsu, Sewickley, PA). Neurons were scored as retrogradely-labeled if the label filled the cell soma and extended into its proximal dendrites and also contained a visible nuclear area devoid of label. The total number of retrogradely-labeled motoneurons whose cut axons had regenerated at least four mm by three weeks post-transection was compared between untreated and optically stimulated groups. The total number of retrogradely-labeled motoneurons that had re-innervated the gastrocnemius muscle by four weeks post-transection was compared between untreated and optically stimulated groups.

### Neuromuscular Junction Analysis

Muscle re-innervation was also assessed by counting the number of re-innervated motor endplates. The number of presynaptic terminals immunoreactive to the synaptic vesicle protein, SV2, was assessed three and four weeks following nerve transection and repair. The re-innervated gastrocnemius muscles were harvested and cryoprotected in sucrose, as described above. Muscles were then sectioned on a cryostat in a horizontal plane at 20 µm thickness, placed on slides, and reacted with an antibody to SV2 (1:50; Developmental Studies Hybridoma Bank) for 72 hours at 4° C, followed by a goat anti-mouse immunoglobulin secondary antibody conjugated to Alexafluor 647 (1:200; Life Technologies D22914), and rhodamine-conjugated α-bungarotoxin (1:1000; Life Technologies B35451). In each muscle studied (20 cases), fluorescent images of 50 motor endplates marked by bungarotoxin binding were obtained and scored for immunoreactivity to SV2 within the boundaries of the endplate, indicating the presence of an overlying neuromuscular synapse. The percentage of re-innervated (SV2+) gastrocnemius motor endplates was compared between treatment groups (untreated and optically treated, each as a percentage of intact).

### Statistical Analyses

For all data derived from histological analyses, slides were coded so that the persons performing the analysis were not aware of the experimental conditions while performing the analysis. A power analysis was conducted based on the variance from a pilot experiment of three thy-1-ChR2/YFP mice that were optically treated and retrogradely labeled at two weeks following nerve repair compared to three untreated mice. According to the power analysis, three to four animals per experimental group was required to reach a significant difference with α = 0.05 and power from 0.75 to 0.97, respectively.

Data are expressed as averages ± SEMs. All statistical tests were performed on the means. For comparisons between two groups, an unpaired student’s t-test was conducted. For data sets including comparisons between three or more groups/times, a one-way analysis of variance (ANOVA) was performed. Paired, post-hoc comparisons were performed using Fisher’s least significant differences (LSD), where appropriate. The alpha for significance was set at p < 0.05 for all comparisons.

## Results

### Anatomical Expression of ChR2/YFP

**Fig 2:**
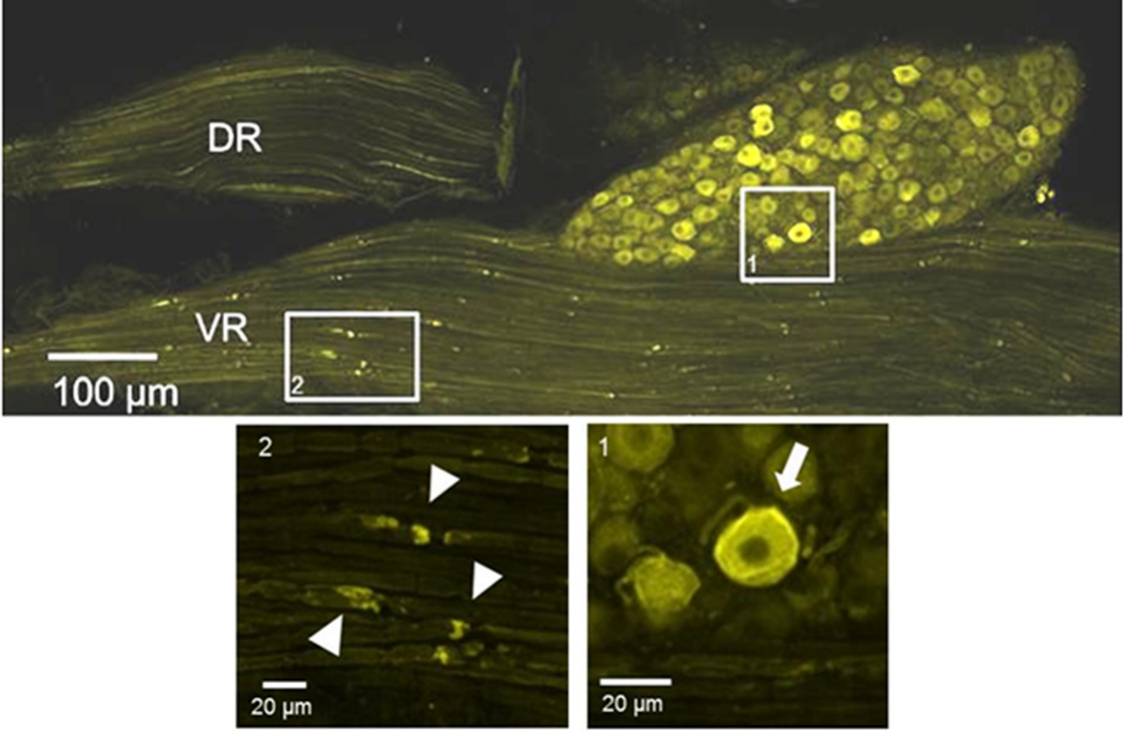
Anatomical expression ChR2/YFP. Image of a whole mount of the L4 dorsal root ganglion, dorsal root, and ventral root from a *Thy1-ChR2/YFP* mouse. Note that a subset of axons in the ventral root (VR) and dorsal root (DR) are ChR2+ as indicated by the presence of YFP in the axons. The insets are a higher magnification of the image marked with a white box. Inset 1: Strong YFP expression was observed in the cell bodies of primary sensory neurons. Inset 2: Small, punctate regions were observed along the motor axons.

Based on our observations from confocal images, we confirmed the previously reported [19] expression of the ChR2/YFP construct along the sciatic nerve in a subset of peripheral axons. This axonal expression is well suited for peripheral nerve stimulation via light for optical neuronal activation. In contrast to dorsal root ganglia, where ChR2/YFP is found in the cell bodies of sensory neurons, in the spinal cord, YFP fluorescence was rarely observed in the somata of motoneurons, even following signal amplification with anti-GFP antibodies. In the spinal roots of thy-1-ChR2/YFP mice, YFP fluorescence is present in motor axons (Fig 2). We interpret these observations to mean that ChR2/YFP is more efficiently trafficked into the axons of motoneurons in these mice (compared to the somata), which precluded an anatomical evaluation of double labeled (ChR2+ and tracer) motoneurons. The expression within the axons was not uniform—small, punctate regions along the axons can be observed in whole mount nerves (Fig 2); this pattern is reminiscent of para-nodal expression described previously in these mice [19]. This irregular axonal expression of YFP is not well suited to the direct measurement of axon profile lengths, such as we have previously reported using thy1/YFP-H mice [20]. Instead, we performed electrophysiological and other anatomical-based experiments to evaluate whether increased neuronal activity is sufficient to enhance axon regeneration.

### Optical Activation Reliably Elicits M Responses

In recordings of optically evoked EMG activity from the gastrocnemius muscle, the amplitude of the M response increased as the intensity of optical activation was slowly increased (Fig 1A). The maximum M response amplitudes evoked with optical stimulation always were smaller than those evoked by electrical stimulation, indicating that not all motor axons were optically active (presumably the ChR2- axons) (Fig 1B). These electrophysiological observations are consistent with anatomical observations that only a subset of the axons in peripheral nerves are YFP+. Based on these observations, we assumed that during an hour of optical treatment of a peripheral nerve (applied just prior to nerve transection and repair), increased neuronal activity occurred in only the subset of axons expressing ChR2. Based on monitoring the M responses, we observed that the muscles (and ChR2) followed the 20 Hz continuous activation during the one hour of optical treatment (Fig 1C).

### Optical Activation Facilitates Axon Regeneration and Muscle Fiber Re-innervation

**Fig 3:**
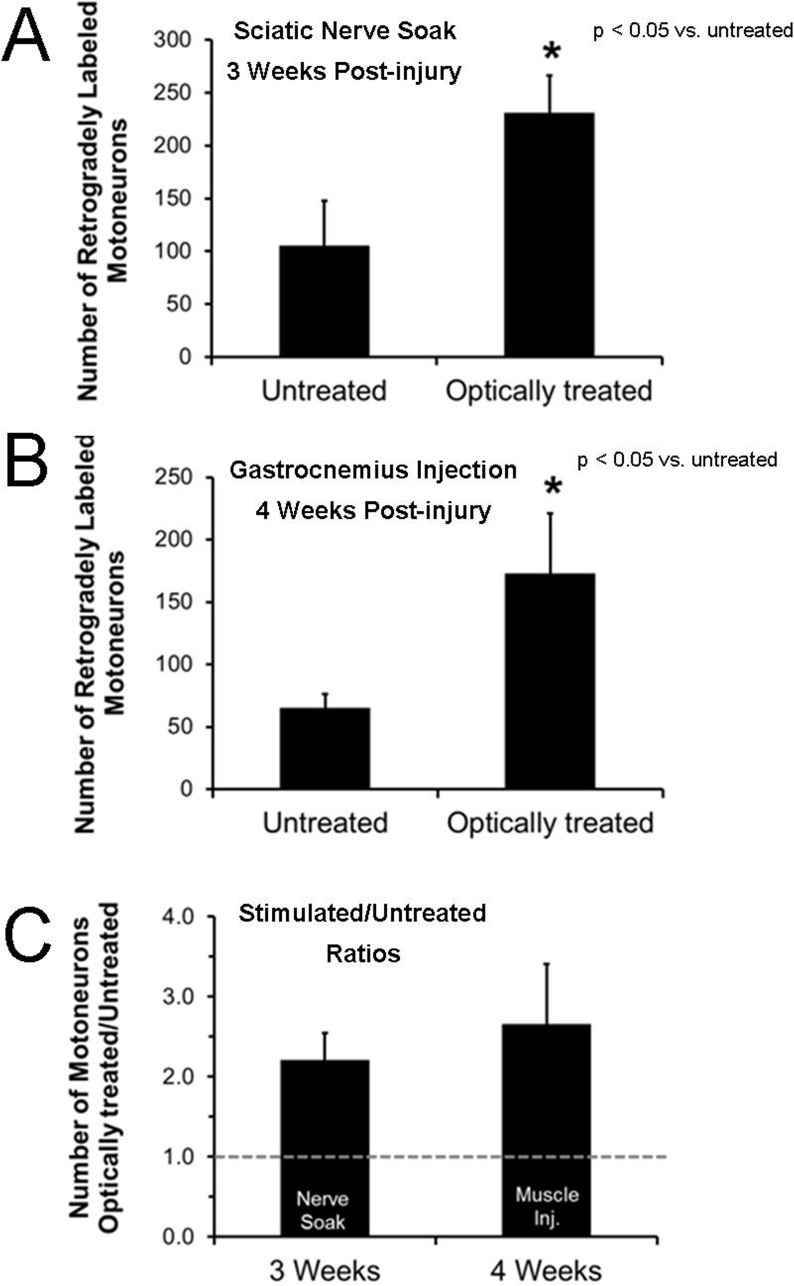
Retrograde labeling to assess axon regeneration. Sciatic nerves were cut and repaired by end-to-end anastomosis. The mean (+SEM) number of motoneurons whose axons had regenerated at least four mm into the distal stump and were labeled by retrograde tracer applied via nerve soak three weeks after transection and repair is shown in panel A. The mean (+SEM) number of motoneurons whose axons had regenerated successfully and had re-innervated the lateral and medial gastrocnemius muscles after tracer injection into muscles four weeks after sciatic nerve transection and repair is shown in panel B. Results from optically treated and untreated mice were compared. C. The ratio of the number of motoneurons in optically treated to untreated mice is shown as mean (+SEM) values. The results from both retrograde labeling methods indicate that more than twice as many motoneurons regenerated following optical treatment.

Axon regeneration and muscle fiber re-innervation were studied using three different assays. We counted the number of motoneurons that had regenerated at least four mm into the distal stump by three weeks after sciatic nerve transection and repair. The average number of motoneurons with axons that had regenerated at least four mm was significantly greater in the optically treated group compared to the untreated group (t(7) 1.89, p < 0.05) (Fig 3A).

By four weeks, most axons have regenerated to at least four mm (even when untreated). Thus, we counted the number of motoneurons that had re-innervated a target muscle by injecting a retrograde label into the medial and lateral gastrocnemius muscles four weeks after sciatic nerve transection and repair, rather than soaking the nerves to these muscles. The average number of motoneurons with axons that had re-innervated the medial and lateral gastrocnemius muscles was significantly greater in the optically treated group compared to the untreated group (*t*(10) 1.81, p < 0.05) (Fig 3B). More than twice as many motoneurons were retrogradely labeled at both three and four weeks following optical treatment compared to untreated controls (Fig 3C).

**Fig 4:**
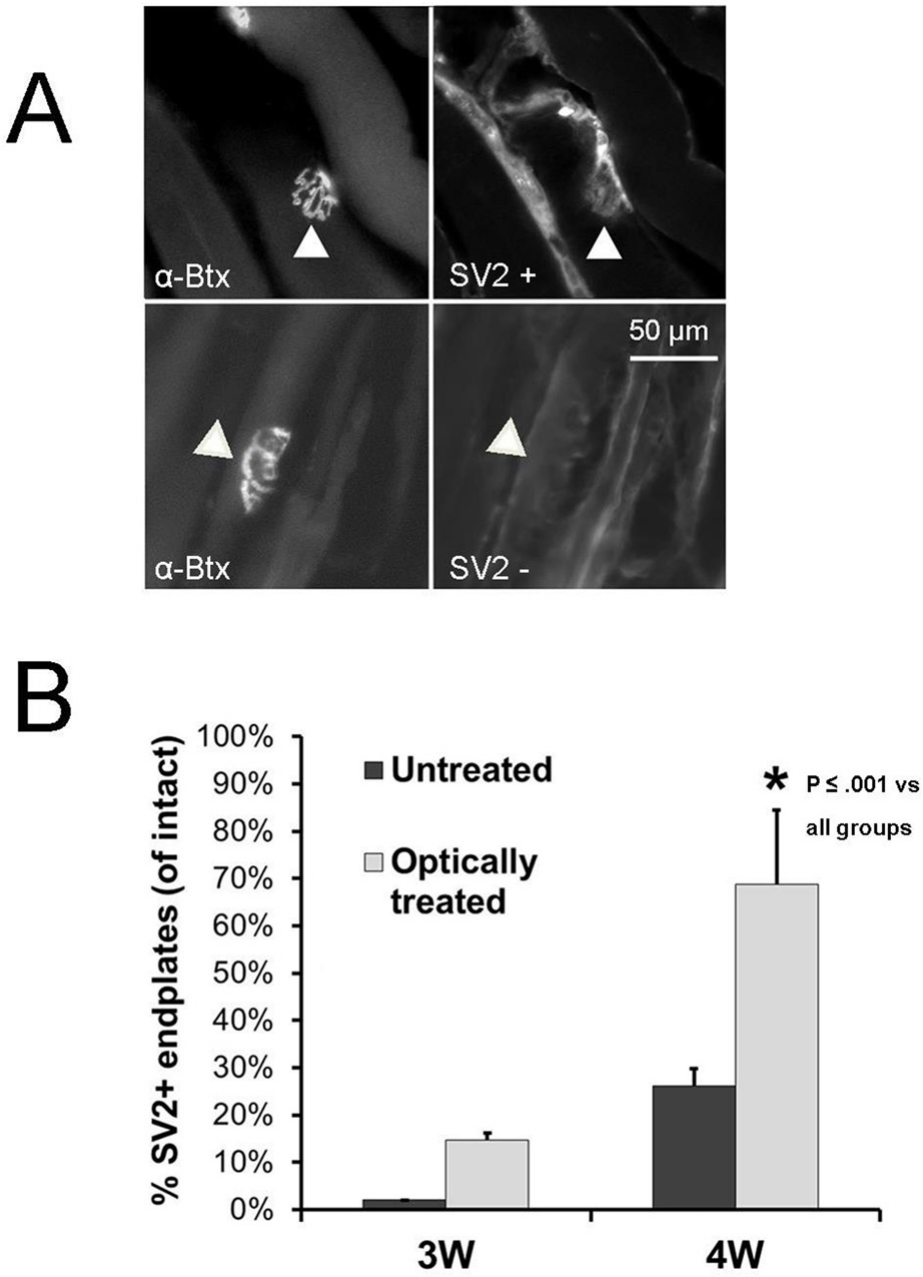
Synaptic Vesicle Protein 2 (SV2) staining to assess muscle reinnervation. A. Using the binding of fluorescent alpha bungarotoxin (α-Btx), two motor endplates are shown (top and bottom left panels). At one endplate, immunoreactivity to SV2 is found (top right, SV2+), but no similar immunoreactivity is found at the second one (bottom right, SV2-). B. Mean (+SEM) percentage of all endplates that were re-innervated (SV2+) at three and four weeks after transection and repair is shown for each group. As expected, significantly more neuromuscular junctions were SV2+ over time when untreated. Optical treatment markedly enhances the number of SV2+ neuromuscular junctions by four weeks after transection and repair * p ≤ 0.001 vs all groups.

Muscle fiber re-innervation also was analyzed by counting the number of gastrocnemius muscle motor endplates where SV2, a marker of presynaptic occupation, was expressed (Fig 4A). In each muscle studied, the proportion of such re-innervated endplates was determined. The omnibus test of the ANOVA of the percentage of SV2+ neuromuscular junctions after each treatment (Fig 4) was significant (F_3, 12_ = 17.82, p < 0.01). The mean percentage of restored neuromuscular junctions in muscle samples following optical treatment was significantly (LSD, *p* ≤ 0.001) higher than all other groups (Fig 4B).

**Fig 5:**
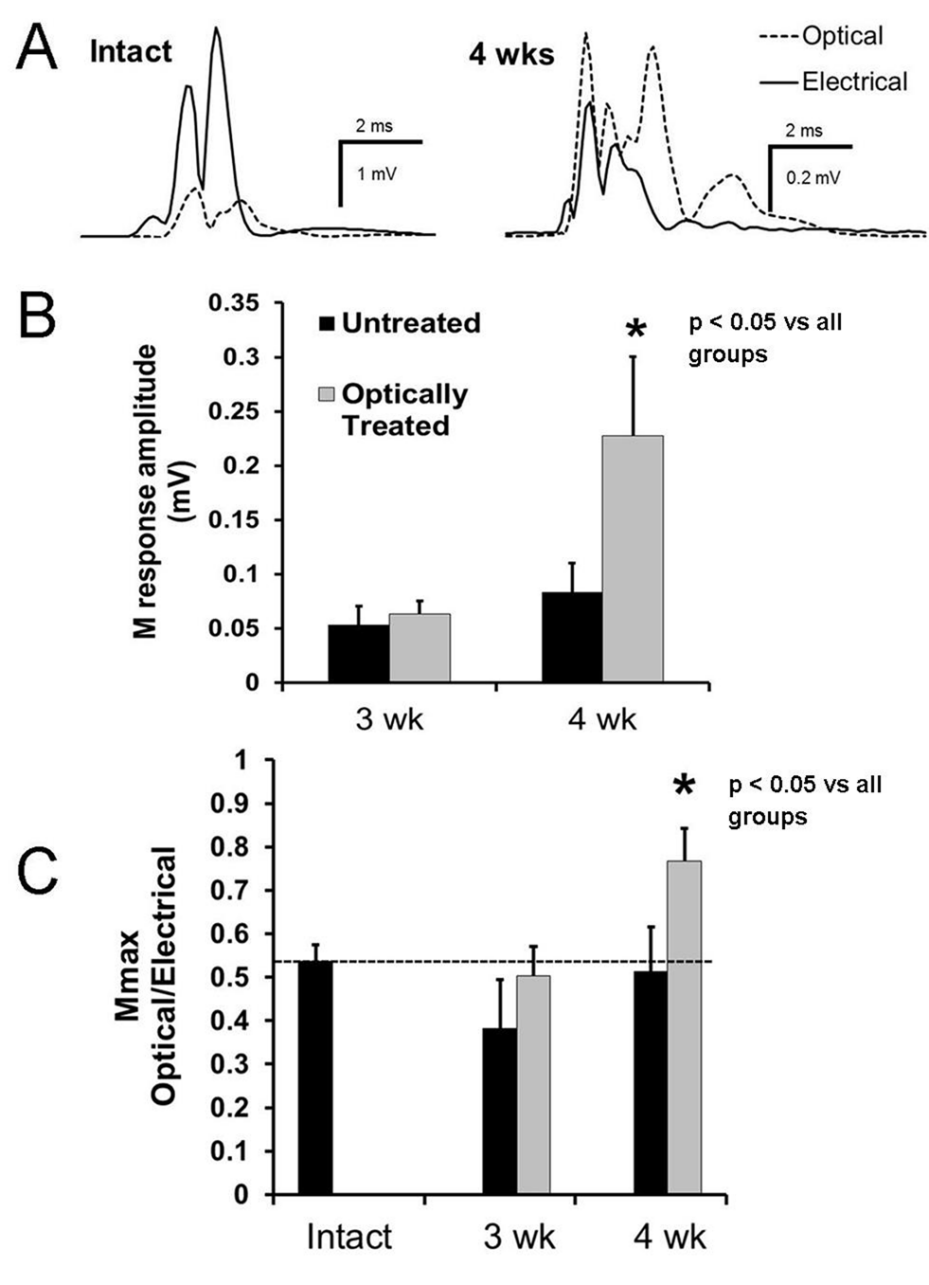
Functional assessment of the gastrocnemius innervation following nerve injury. M responses elicited by optical and electrical nerve stimulation were measured three and four weeks after transection and repair of the sciatic nerve. A. Examples of electrically evoked (solid traces) and optically evoked (dashed traces) M responses recorded from the gastrocnemius muscle of a thy-1-ChR2/YFP mouse. Recordings were made prior to (Intact) and four weeks after transection and surgical repair of the sciatic nerve, followed by one hour of optical stimulation. B. Mean (+SEM) M response amplitudes, evoked optically from the gastrocnemius muscles at three and four weeks after sciatic nerve transection and repair are shown for both: optically treated and untreated animals. * p < 0.05 vs all other groups. C. The mean (+SEM) ratio of optically evoked to electrically evoked M responses recorded from gastrocnemius muscles of optically treated and untreated mice is shown. Measurements presented were made in Intact animals and three or four weeks after transection and repair of the sciatic nerve. The horizontal dashed line is placed at the mean ratio found in intact mice. * p < 0.05 vs all other groups.

Finally, axon regeneration and muscle fiber re-innervation were assessed via analysis of evoked EMG responses in the re-innervated muscles. Direct muscle (M) responses were evoked both optically and electrically prior to and at three and four weeks after nerve transection and repair (Fig 5A). The omnibus tests of the ANOVA of the amplitudes of the optically- and electrically-evoked M responses were significant (F_3, 42_ = 4.968, p < 0.01; F_3, 42_ = 3.522, p < 0.05, respectively). By four weeks post-injury, the optically- and electrically-evoked M response amplitudes were significantly larger in the optically treated group than in the untreated group (LSD, both p < 0.05), indicating that more fibers in the gastrocnemius muscles had been re-innervated by ChR2+ axons as a result of the single (one hour) optical treatment (Fig 5B).

If the amplitudes of the evoked M responses were expressed as ratios (optical/electrical), the selectivity of the optical treatment is observed (Fig 5C). Only a subset of motor axons in these mice are ChR2+, and this limited expression is reflected in the ratio of optical/electrical M response amplitudes (Fig 5A left panel). In our experiments, the mean ratio measured in intact mice is 0.53 ± 0.037 (n = 38 unoperated controls). The omnibus test of the ANOVA of the optical/electrical ratio was significant (F_4, 79_ = 2.861, p < 0.05). The ratios found for untreated mice at both the three and four week times examined and for the optically treated mice at three weeks after nerve injury are not significantly different. However, in optically treated mice at four weeks after nerve injury, this ratio (0.77 ± 0.075, n = 10) is significantly greater than found in intact mice (LSD, p < 0.05) or untreated controls (LSD, p < 0.05) (Fig 5A, right panel, Fig 5C).

## Discussion

In 1983, Nix and Hopf demonstrated that low frequency electrical stimulation could enhance motor functional recovery following nerve crush in rabbits [21]. Since then, research efforts have focused on the mechanisms and effects of brief electrical stimulation [12]. The rationale supporting this intervention is that electrical stimulation directly increases neuronal activity and could be easily applied by surgeons during repair. For example, in an experiment utilizing brief electrical treatment (1 hour), Gordon and colleagues demonstrated that activation of the axotomized neuron cell bodies, and not stimulation of the muscle, was *necessary* for the enhancement of axon regeneration by electrical stimulation (9). Other studies by our lab and others have elucidated some of the mechanisms responsible for this enhanced regeneration [22–25].

Optogenetic-based experiments have enormous potential to answer novel questions regarding the role of neuronal activity during central or peripheral nervous system injuries. Yet, few animal studies have been performed [16, 26–29] in this broad field despite the establishment of optical neuronal control over a decade previously [30–32]. In this study, we present the first report utilizing optical activation to enhance motor axon regeneration *in vivo* following peripheral nerve transection. We tested the hypothesis that neuronal activity alone is sufficient to enhance axon regeneration. Based on several outcome measures in *thy-1-ChR2/YFP* mice, we conclude that brief (one hour) optical activation enhanced axon regeneration.

We assessed whether brief optical stimulation would result in functional gains and assessed parameters that contribute to muscle function. We quantified the number of presynaptic terminals re-innervating motor endplates in the gastrocnemius muscle in optically treated or untreated mice. By one month post-transection, optical treatment resulted in significantly more re-formed neuromuscular junctions compared to untreated controls. Simultaneously, the number of motoneurons that could be retrogradely labeled from the target muscle was significantly greater in the optical treatment group. Two weeks post-transection, the mean number of retrogradely-labeled motoneurons following one hour of optical treatment (217 ± 44) is not significantly different from one hour of electrical stimulation (198 ± 72) or 2 weeks of exercise (181 ± 34) (after [33]). Thus, the acute effect of optical stimulation is to effectively promote axon regeneration across the injury site.

Finally, we assessed the M response amplitudes in optically treated and untreated animals. The mean maximal M response amplitudes were significantly greater in optically treated animals at four weeks than in untreated controls. Furthermore, the mean maximal M response amplitudes in optically treated animals were not significantly different from those of wild type mice that underwent two weeks of exercise (t(8) 1.05, n.s.). Together, these observations are consistent with our hypothesis that increasing neuronal activity is sufficient to enhance axon regeneration and muscle re-innervation following nerve transection and repair.

We previously demonstrated that the mechanism of activity-based therapies requires neuronal brain derived neurotrophic factor (BDNF) and also androgen receptor signaling [23, 34–36], although the precise interaction between these two pathways is not clear at this time. It is possible that neurotrophin or androgen released by the activated neurons could facilitate or enhance the regeneration of neighboring axons either by direct neurotrophic support or through the recruitment/alteration of other supporting cells (e.g. Schwann cells, microglia, and astrocytes) that may affect the local environment around the motor nucleus or around the elongating axons. In a recent study using explant cultures, optical treatment of cultured dorsal root ganglion neurons facilitated directional neurite outgrowth of both activated and inactive neurons [37]. These authors attributed the non-specific enhancement of neurite growth to a paracrine effect of the release of growth factors by optically activated neurons *in vitro*.

We suggest that optical treatment enhanced the regeneration of motoneurons that were ChR2+ selectively. In our experiments, we recorded both optically and electrically evoked M response amplitudes. When expressed as a ratio (optical/electrical), a ratio of 1.00 would indicate that all of the motor axons in the nerve were ChR2+, and a ratio of 0.00 would indicate that none of the motor axons in the nerve could be activated by light (ChR2-). Based on the results of our experiments, we suggest that approximately half of all motor axons innervating the gastrocnemius muscle in intact *thy-1-ChR2/YFP* mice are optically activatable (ChR2+) (Fig 5C). In untreated animals at one month post-transection, this ratio is not significantly different from intact controls, suggesting that ChR2+ and ChR2- motor axons are about equally successful in regenerating and re-innervating gastrocnemius muscle fibers. In contrast, one month after one hour of optical activation is applied as a treatment at the time of nerve repair, this ratio is more than 50% greater than controls and not significantly different from 1.0, meaning that the overwhelming majority of axons that had regenerated successfully and re-innervated the target muscle at that time were activatable by light–i.e. the axons that were treated. We interpret these results to mean that optical treatment provided a selective regenerative advantage to the ChR2+ axons.

In this study, we focused on motor axon regeneration and muscle reinnervation. However, it should be noted that ChR2+ primary afferent neurons were also stimulated during the optical treatment, and their possible contribution to motor axon regeneration is unclear. In future studies, it will be interesting to determine the potential effect of primary afferent neurons on long-term functional recovery.

We find our results consistent with the notion that brief activation of a subset of axons in a nerve does not provide substantial benefit to neighboring, unstimulated axons *in vivo*. This conclusion is supported by our previous studies utilizing mice in which a subset of neurons were made null for the gene for BDNF [34, 38]. In these mice, two weeks of treadmill exercise failed to enhance axon regeneration and failed to restore the synaptic inputs onto motoneurons lacking BDNF. Even though the majority of neurons in this mouse were wild-type, the BDNF (and other growth factors) secreted from these wild-type neurons could not rescue the neighboring BDNF knock-out neurons/axons *in vivo*.

In summary, activity-based therapies are novel and exciting treatments for patients with peripheral nerve injuries because they are effective and easily applied. There are many aspects of these therapies that have not been thoroughly researched, leaving clinicians with questions about how to most effectively implement these treatments. We hypothesize that *the* critical feature of exercise is that it increases the activity of axotomized neurons and stimulates the regeneration of their axons. In this study, we optically activated motor axons in transected sciatic nerves and assayed their recovery, both anatomically and functionally. Brief neuronal activity was sufficient to enhance axon elongation and muscle re-innervation. The nature of, and limits to, the effect of increased activity are mostly unknown, especially when considering the impact of long-term functional recovery in humans. Our interpretation of the findings from this study is that the previously implicated mechanisms of neurotrophic factor upregulation and androgen receptor signaling are limited to the neurons that are directly activated.

## References

[1] Noble J, Munro CA, Prasad VS, Midha R. Analysis of upper and lower extremity peripheral nerve injuries in a population of patients with multiple injuries. The Journal of trauma. 1998;45(1):116–22. Epub 1998/07/29. . 9680023 10.1097/00005373-199807000-00025

[2] Taylor CA, Braza D, Rice JB, Dillingham T. The incidence of peripheral nerve injury in extremity trauma. American journal of physical medicine & rehabilitation / Association of Academic Physiatrists. 2008;87(5):381–5. Epub 2008/03/13. 10.1097/PHM.0b013e31815e6370 . 18334923

[3] Isaacs J. Treatment of acute peripheral nerve injuries: current concepts. The Journal of hand surgery. 2010;35(3):491–7; quiz 8. Epub 2010/02/09. 10.1016/j.jhsa.2009.12.009 . 20138714

[4] Scholz T, Krichevsky A, Sumarto A, Jaffurs D, Wirth GA, Paydar K, et al Peripheral nerve injuries: an international survey of current treatments and future perspectives. Journal of reconstructive microsurgery. 2009;25(6):339–44. Epub 2009/03/21. 10.1055/s-0029-1215529 . 19301234

[5] Frostick SP, Yin Q, Kemp GJ. Schwann cells, neurotrophic factors, and peripheral nerve regeneration. Microsurgery. 1998;18(7):397–405. Epub 1999/01/08. . 9880154 10.1002/(sici)1098-2752(1998)18:7<397::aid-micr2>3.0.co;2-f

[6] Boeltz T, Ireland M, Mathis K, Nicolini J, Poplavski K, Rose SJ, et al Effects of treadmill training on functional recovery following peripheral nerve injury in rats. Journal of neurophysiology. 2013;109(11):2645–57. Epub 2013/03/08. 10.1152/jn.00946.2012 23468390 PMC3680800

[7] Udina E, Puigdemasa A, Navarro X. Passive and active exercise improve regeneration and muscle reinnervation after peripheral nerve injury in the rat. Muscle & nerve. 2011;43(4):500–9. Epub 2011/02/10. 10.1002/mus.21912 . 21305568

[8] Sabatier MJ, English AW. Pathways Mediating Activity-Induced Enhancement of Recovery from Peripheral Nerve Injury. Exercise and sport sciences reviews. 2015 Epub 2015/04/24. 10.1249/JES.0000000000000047 . 25906422 PMC4470799

[9] Al-Majed AA, Neumann CM, Brushart TM, Gordon T. Brief electrical stimulation promotes the speed and accuracy of motor axonal regeneration. The Journal of neuroscience: the official journal of the Society for Neuroscience. 2000;20(7):2602–8. Epub 2000/03/24. . 10729340 10.1523/JNEUROSCI.20-07-02602.2000 PMC6772244

[10] Gordon T, Amirjani N, Edwards DC, Chan KM. Brief post-surgical electrical stimulation accelerates axon regeneration and muscle reinnervation without affecting the functional measures in carpal tunnel syndrome patients. Experimental neurology. 2010;223(1):192–202. Epub 2009/10/06. 10.1016/j.expneurol.2009.09.020 . 19800329

[11] Gordon T, Chan KM, Sulaiman OA, Udina E, Amirjani N, Brushart TM. Accelerating axon growth to overcome limitations in functional recovery after peripheral nerve injury. Neurosurgery. 2009;65(4 Suppl):A132–44. Epub 2009/12/16. 10.1227/01.NEU.0000335650.09473.D3 19927058

[12] Gordon T, English AW. Strategies to promote peripheral nerve regeneration: electrical stimulation and/or exercise. The European journal of neuroscience. 2015 Epub 2015/06/30. 10.1111/ejn.13005 . 26121368 PMC4695319

[13] Arenkiel BR, Peca J, Davison IG, Feliciano C, Deisseroth K, Augustine GJ, et al In vivo light-induced activation of neural circuitry in transgenic mice expressing channelrhodopsin-2. Neuron. 2007;54(2):205–18. Epub 2007/04/20. 10.1016/j.neuron.2007.03.005 17442243 PMC3634585

[14] de Vries J, Menovsky T, van Gulik S, Wesseling P. Histological effects of fibrin glue on nervous tissue: a safety study in rats. Surgical neurology. 2002;57(6):415–22; discussion 22. Epub 2002/08/15. . 12176207 10.1016/s0090-3019(02)00736-x

[15] MacGillivray TE. Fibrin sealants and glues. Journal of cardiac surgery. 2003;18(6):480–5. Epub 2004/03/03. . 14992096 10.1046/j.0886-0440.2003.02073.x

[16] Towne C, Montgomery KL, Iyer SM, Deisseroth K, Delp SL. Optogenetic control of targeted peripheral axons in freely moving animals. PloS one. 2013;8(8):e72691 Epub 2013/08/31. 10.1371/journal.pone.0072691 23991144 PMC3749160

[17] English AW, Cucoranu D, Mulligan A, Sabatier M. Treadmill training enhances axon regeneration in injured mouse peripheral nerves without increased loss of topographic specificity. The Journal of comparative neurology. 2009;517(2):245–55. Epub 2009/09/05. 10.1002/cne.22149 19731339 PMC2804895

[18] McLean IW, Nakane PK. Periodate-lysine-paraformaldehyde fixative. A new fixation for immunoelectron microscopy. The journal of histochemistry and cytochemistry: official journal of the Histochemistry Society. 1974;22(12):1077–83. Epub 1974/12/01. . 4374474 10.1177/22.12.1077

[19] Llewellyn ME, Thompson KR, Deisseroth K, Delp SL. Orderly recruitment of motor units under optical control in vivo. Nature medicine. 2010;16(10):1161–5. Epub 2010/09/28. 10.1038/nm.2228 . 20871612 PMC5839640

[20] Groves ML, McKeon R, Werner E, Nagarsheth M, Meador W, English AW. Axon regeneration in peripheral nerves is enhanced by proteoglycan degradation. Experimental neurology. 2005;195(2):278–92. Epub 2005/06/14. 10.1016/j.expneurol.2005.04.007 . 15950970

[21] Nix WA, Hopf HC. Electrical stimulation of regenerating nerve and its effect on motor recovery. Brain research. 1983;272(1):21–5. Epub 1983/08/01. . 6616196 10.1016/0006-8993(83)90360-8

[22] Tam SL, Archibald V, Tyreman N, Gordon T. Tetrodotoxin prevents motor unit enlargement after partial denervation in rat hindlimb muscles. The Journal of physiology. 2002;543(Pt 2):655–63. Epub 2002/09/03. 12205197 10.1113/jphysiol.2001.012982 PMC2290525

[23] Thompson NJ, Sengelaub DR, English AW. Enhancement of peripheral nerve regeneration due to treadmill training and electrical stimulation is dependent on androgen receptor signaling. Developmental neurobiology. 2014;74(5):531–40. Epub 2013/12/03. 10.1002/dneu.22147 . 24293191 PMC4730387

[24] English AW, Liu K, Nicolini JM, Mulligan AM, Ye K. Small-molecule trkB agonists promote axon regeneration in cut peripheral nerves. Proceedings of the National Academy of Sciences of the United States of America. 2013;110(40):16217–22. Epub 2013/09/18. 10.1073/pnas.1303646110 24043773 PMC3791704

[25] Sharma N, Marzo SJ, Jones KJ, Foecking EM. Electrical stimulation and testosterone differentially enhance expression of regeneration-associated genes. Experimental neurology. 2010;223(1):183–91. Epub 2009/05/12. 10.1016/j.expneurol.2009.04.031 . 19427307

[26] Bryson JB, Machado CB, Crossley M, Stevenson D, Bros-Facer V, Burrone J, et al Optical control of muscle function by transplantation of stem cell-derived motor neurons in mice. Science. 2014;344(6179):94–7. Epub 2014/04/05. 10.1126/science.1248523 . 24700859 PMC5947756

[27] Daou I, Tuttle AH, Longo G, Wieskopf JS, Bonin RP, Ase AR, et al Remote optogenetic activation and sensitization of pain pathways in freely moving mice. The Journal of neuroscience: the official journal of the Society for Neuroscience. 2013;33(47):18631–40. Epub 2013/11/22. 10.1523/JNEUROSCI.2424-13.2013 . 24259584 PMC6618811

[28] Boada MD, Martin TJ, Peters CM, Hayashida K, Harris MH, Houle TT, et al Fast-conducting mechanoreceptors contribute to withdrawal behavior in normal and nerve injured rats. Pain. 2014;155(12):2646–55. Epub 2014/10/01. 10.1016/j.pain.2014.09.030 25267211 PMC4374598

[29] Alilain WJ, Li X, Horn KP, Dhingra R, Dick TE, Herlitze S, et al Light-induced rescue of breathing after spinal cord injury. The Journal of neuroscience: the official journal of the Society for Neuroscience. 2008;28(46):11862–70. Epub 2008/11/14. 10.1523/JNEUROSCI.3378-08.2008 19005051 PMC2615537

[30] Zemelman BV, Lee GA, Ng M, Miesenbock G. Selective photostimulation of genetically chARGed neurons. Neuron. 2002;33(1):15–22. Epub 2002/01/10. . 11779476 10.1016/s0896-6273(01)00574-8

[31] Banghart M, Borges K, Isacoff E, Trauner D, Kramer RH. Light-activated ion channels for remote control of neuronal firing. Nature neuroscience. 2004;7(12):1381–6. Epub 2004/11/24. 10.1038/nn1356 15558062 PMC1447674

[32] Nagel G, Szellas T, Huhn W, Kateriya S, Adeishvili N, Berthold P, et al Channelrhodopsin-2, a directly light-gated cation-selective membrane channel. Proceedings of the National Academy of Sciences of the United States of America. 2003;100(24):13940–5. Epub 2003/11/15. 10.1073/pnas.1936192100 14615590 PMC283525

[33] English AW. Enhancing axon regeneration in peripheral nerves also increases functionally inappropriate reinnervation of targets. The Journal of comparative neurology. 2005;490(4):427–41. Epub 2005/08/30. 10.1002/cne.20678 . 16127712

[34] Wilhelm JC, Xu M, Cucoranu D, Chmielewski S, Holmes T, Lau KS, et al Cooperative roles of BDNF expression in neurons and Schwann cells are modulated by exercise to facilitate nerve regeneration. The Journal of neuroscience: the official journal of the Society for Neuroscience. 2012;32(14):5002–9. Epub 2012/04/12. 10.1523/JNEUROSCI.1411-11.2012 22492055 PMC3382119

[35] Liu C, Ward PJ, English AW. The effects of exercise on synaptic stripping require androgen receptor signaling. PloS one. 2014;9(6):e98633 Epub 2014/06/03. 10.1371/journal.pone.0098633 24887087 PMC4041790

[36] English AW, Wilhelm JC, Ward PJ. Exercise, neurotrophins, and axon regeneration in the PNS. Physiology. 2014;29(6):437–45. Epub 2014/11/05. 10.1152/physiol.00028.2014 25362637 PMC4280152

[37] Park S, Koppes RA, Froriep UP, Jia X, Achyuta AK, McLaughlin BL, et al Optogenetic control of nerve growth. Scientific reports. 2015;5:9669 Epub 2015/05/20. 10.1038/srep09669 . 25982506 PMC4434892

[38] Krakowiak J, Liu C, Papudesu C, Ward PJ, Wilhelm JC, English AW. Neuronal BDNF signaling is necessary for the effects of treadmill exercise on synaptic stripping of axotomized motoneurons. Neural plasticity. 2015;2015:392591 Epub 2015/04/29. 10.1155/2015/392591 25918648 PMC4397030

